# The Impact of COVID-19 on the Functioning of a Neurosurgical Service at a Tertiary Institute in a Low-Resource Setting

**DOI:** 10.7759/cureus.22294

**Published:** 2022-02-16

**Authors:** Panduranga Seetahal-Maraj, Sharies R Arjoon, Narindra Ramnarine, Dylan Thomas, Phillip St Louis

**Affiliations:** 1 Neurosurgery, San Fernando General Hospital, San Fernando, TTO; 2 Clinical Surgical Sciences, The University of the West Indies, St. Augustine, TTO

**Keywords:** cancellation elective surgical operations, trinidad and tobago, neurosurgery in developing countries, low-resource setting, covid-19 pandemic

## Abstract

Objective

During the COVID-19 pandemic, certain precautionary measures were implemented to limit its effect, including the cancellation of clinics and elective surgical lists. To determine the impact, if any, of the pandemic on the running of a neurosurgical service, an audit was performed on the volume of referrals, admissions and type of surgeries performed at a tertiary institute in a low-resource setting.

Methods

An audit of the neurosurgical department’s database was performed on the number of referrals, admissions, surgical procedures, and types of procedures done at the San Fernando General Hospital. This was divided into two 15-month periods, pre-pandemic (January 1, 2019 to March 31, 2020) and intra-pandemic (April 1, 2020 - June 30, 2021).

Results

During the pre-pandemic period (January 1, 2019 - March 31, 2020), 2,597 patients were referred to the service and 309 major procedures were performed. Two thousand and forty-two patients were referred during the intra-pandemic (April 4, 2020 - June 30, 2021) period, with 354 surgeries performed.

More external ventricular drains (29 vs 50), craniotomies for trauma (73 vs 98), anterior cervical fusion (42 vs 47), lumbar fusions (9 vs 12), ventriculo-peritoneal shunts (16 vs 19) and aneurysm clipping (10 vs 13) were done during the intra-pandemic period.

Conclusion

Although elective surgical procedures and clinics were reduced, the number of patients seen and total procedures performed did not vary significantly. The neurosurgical pathology encountered remained constant during the pandemic. This shows the importance of maintaining a fully functional neurosurgical service, as we continue to adapt to the COVID-19 pandemic.

## Introduction

The COVID-19 pandemic in Trinidad and Tobago created certain challenges in managing the on-call referrals and admissions, surgical procedures and clinics for the neurosurgical department of San Fernando General Hospital (SFGH). While there were periodical shutdowns of the clinics and elective surgeries due to surges in COVID-19 positive patients, the number of neurosurgical referrals and procedures are done appeared unchanged.

Our aim was to audit and analyse the numbers pre-pandemic and intra-pandemic, to ascertain if there was any impact on the care and service provided. If there was a change in the operative volume and type of procedure performed, this would allow for better resource allocation and equipment ordering during this pandemic.

## Materials and methods

The neurosurgical department utilises a spreadsheet database, and all referrals and admissions are logged by the on-call officers. The surgical logbook housed in the operating theatre includes all neurosurgical procedures performed. These sources were examined to collate the numbers of emergency/ward referrals seen, ward admissions and surgical cases done. It was carried out during two timeframes - period A, January 1, 2019 - March 31, 2020 (pre-pandemic) and period B, April 1, 2020 - June 30, 2021 (intra-pandemic).

## Results

In period A, there were 2,145 emergency department referrals and 452 ward referrals made to the neurosurgical service, totalling 2,597 patients during the pre-pandemic phase. In period B, the emergency department made 1830 referrals and ward referrals numbered 452, totalling 2,042 patients. Primary admissions numbered 760 during period A, with 415 patients being comanaged with another department. During period B, 1,251 patients were primarily neurosurgical, with 568 patients being comanaged. (Tables 1, 2).

**Table 1:** Pre-pandemic (January 1, 2019 - March 31, 2020) numbers of referrals and admissions to a neurosurgical service.

Month	A&E referrals	Ward referrals	Primary admissions	Comanaged patients	Total referrals
January 2019	118	10	29	30	128
February 2019	147	27	45	32	174
March 2019	182	22	48	52	204
April 2019	147	25	56	25	172
May 2019	153	30	59	25	183
June 2019	149	27	61	27	179
July 2019	135	28	50	19	163
August 2019	137	42	66	16	179
September 2019	147	33	61	30	180
October 2019	138	52	41	10	190
November 2019	154	56	43	21	210
December 2019	156	61	56	30	217
January 2020	139	9	38	51	148
February 2020	137	7	67	31	144
March 2020	106	23	40	16	129
Totals	2145	452	760	415	2597

**Table 2:** Intra-pandemic (April 1, 2020 - June 30, 2021) numbers of referrals and admissions.

Month	A&E referrals	Ward referrals	Primary admissions	Comanaged patients	Total referrals
April 2020	96	7	55	39	103
May 2020	126	12	80	54	138
June 2020	141	6	82	36	147
July 2020	136	25	113	44	161
August 2020	147	2	102	43	149
September 2020	138	5	90	38	143
October 2020	125	6	86	28	131
November 2020	118	8	81	39	126
December 2020	104	10	73	51	114
January 2021	108	25	71	36	133
February 2021	103	21	72	28	124
March 2021	125	30	91	34	155
April 2021	122	15	89	32	137
May 2021	120	21	81	27	141
June 2021	121	19	85	39	140
Total	1830	212	1251	568	2042

Surgical volume for period A was 309 major procedures, whereas 354 were done during period B (Table 3). Comparing periods A to B, there were higher numbers for certain procedures during the pandemic. These included external ventricular drains (29 vs 50), craniotomies for trauma (73 vs 98), anterior cervical fusion (42 vs 47), lumbar fusions (9 vs 12), ventriculoperitoneal shunts (16 vs 19) and aneurysm clipping (10 vs 13). 

**Table 3:** Surgical procedures and volume per procedure for both periods.

Procedure	Period A (January 1, 2019 - March 31, 2020)	Period B (April 1, 2020 - June 30, 2021)
External ventricular drain	29	50
Decompressive craniectomy	22	6
Aneurysm clipping	10	13
Tumour debulking	31	27
Cranioplasty	2	5
Transsphenoidal resection	1	7
Craniotomy for trauma	73	98
Ventriculo-peritoneal shunt	16	19
Other cranial procedure	11	2
Anterior cervical fusion	42	47
Posterior cervical fusion	6	5
Other cervical spinal procedure	3	6
Lumbar laminectomy	47	43
Thoracolumbar corpectomy	3	4
Lumbar fusion	9	12
Other thoracolumbar spinal procedure	4	10
Total	309	354

## Discussion

Neurosurgical services have evolved and improved over the years, with more pathology being deemed treatable. Increases in both the numbers of emergency referrals and lowered thresholds for referrals have been documented [1]. The COVID-19 pandemic has affected the running of neurosurgical services worldwide, with varying effects seen. Some services have noted no change in their volume [2], or an increase in emergency procedures performed [3]. Other centres have seen a reduction in procedures and admissions [4-6].

In Trinidad and Tobago, this pandemic resulted in the creation of a parallel healthcare system to manage affected patients, in addition to the existing government-funded public healthcare model. This is under the purview of specific regional health authorities, of which the San Fernando General Hospital is the main tertiary institute for the South-West Regional Health Authority. Neurosurgical services for this region are only provided at this institution, and it caters to 600,000 paediatric and adult populations [7].

No data were previously collated to assess the volumes treated by our service, and during the pandemic we expected that volumes may have been altered. This was due to the intermittent reduction/cancelling of outpatient clinics and elective operating lists, reassignment of staff from the department to assist in COVID-19 hospitals and public health measures including a state of emergency. Neurosurgical emergencies cannot be deferred, as they tend to result in poorer outcomes [2], and we anticipated that the overall volume would remain the same. The auditing of two 15-month periods to assess the output of the department both established data and will allow future analyses.

The impact of COVID-19 on our neurosurgical service 

There were 555 fewer total referrals to us during the intra-pandemic period; however, there were 509 more primary admissions to the service. This may indicate that more of the referrals required procedures and possibly a lower threshold for admission. There were significantly more craniotomies for trauma and EVDs performed during the pandemic. Spinal cases did not significantly vary in volume.

Factors affecting volumes

In Trinidad, overall shortages of healthcare workers, including those with particular skills and their subsequent distribution, contribute to challenges in the public healthcare system [8]. This resulted in staff burnout and may have led to delays in patient care.

At our institute, there are currently three consultant surgeons in the neurosurgical department - two neurosurgeons and an orthopaedic spine surgeon. The addition of a fellowship-trained vascular neurosurgeon in September 2018 has increased the overall volume of general neurosurgical and vascular procedures performed. In July 2020, the addition of a fellowship-trained spine surgeon further boosted the number of spinal surgeries that could be done weekly.

Another important factor is the availability of neurosurgical equipment for the performance of procedures. Neurosurgery is a heavily equipment-dependent speciality, and certain procedures are not possible without the required equipment. Difficulty in procuring this equipment as a result of delays in shipping and customs due to COVID-19 would have led to longer waiting times for surgeries.

Limitations

The databases used rely on individual input by the officers, and human error may result in some entries not being logged. This will affect the true number of referrals. Also, this data was collected from a single institution, and may not reflect the situation at other hospitals.

The lack of an electronic medical record system hinders data collection. Reliable and easy access to disease coding for every admission would facilitate a sub-group analysis of the types of referrals encountered (for example, hydrocephalus, chronic subdural haematomas, cauda equina syndrome, etc.).

## Conclusions

The COVID-19 pandemic and the resultant state of emergency in Trinidad and Tobago lead to challenges throughout the public health care system. Despite certain restrictions, the neurosurgical service at our institution had an increase in primary admissions and in certain surgical procedures. This was due to the nature of neurosurgical emergencies and the need for prompt management to prevent neurological death or decline. This also highlights the need to maintain a fully functional neurosurgical department, despite being in a resource-limited setting.

## References

[1] Spencer RJ, Amer S, St George EJ (2021). A retrospective analysis of emergency referrals and admissions to a regional neurosurgical centre 2016-2018. Br J Neurosurg.

[2] Hecht N, Wessels L, Werft FO, Schneider UC, Czabanka M, Vajkoczy P (2020). Need for ensuring care for neuro-emergencies-lessons learned from the COVID-19 pandemic. Acta Neurochir (Wien).

[3] Ashkan K, Jung J, Velicu AM (2021). Neurosurgery and coronavirus: impact and challenges-lessons learnt from the first wave of a global pandemic. Acta Neurochir (Wien).

[4] Saad H, Alawieh A, Oyesiku N, Barrow DL, Olson J (2020). Sheltered neurosurgery during COVID-19: the Emory experience. World Neurosurg.

[5] Jayakumar N, Kennion O, Villabona AR, Paranathala M, Holliman D (2020). Neurosurgical referral patterns during the coronavirus disease 2019 pandemic: a United Kingdom experience. World Neurosurg.

[6] Koester SW, Catapano JS, Ma KL (2021). COVID-19 and neurosurgery consultation call volume at a single large tertiary center with a propensity-adjusted analysis. World Neurosurg.

[7] (2021). San Fernando General Hospital. http://www.swrha.co.tt/content/about-swrha.

[8] Hunte SA, Pierre K, St Rose R, Simeon DT (2020). Health systems’ resilience: COVID-19 response in Trinidad and Tobago. Am J Trop Med Hyg.

